# How socioeconomic status, social capital and functional independence are associated with subjective wellbeing among older Indian adults? A structural equation modeling analysis

**DOI:** 10.1186/s12889-022-14215-4

**Published:** 2022-09-30

**Authors:** T. Muhammad, Pradeep Kumar, Shobhit Srivastava

**Affiliations:** International Institute for Population Sciences, Mumbai, Maharashtra India 400088

**Keywords:** Socioeconomic status, Functional independence, Social capital, SWB, Older adults

## Abstract

**Background:**

Subjective well-being (SWB) is of particular interest among gerontologists and health researchers with important implications for interventions especially in poor-resource settings. This study aimed to explore the possible pathways from socioeconomic status (SES), functional independence and social capital towards SWB among older adults in India.

**Methods:**

Cross-sectional data from the “Building a Knowledge Base on Population Aging in India” (BKPAI) survey with a total sample of 9231 older adults aged 60 years and above were used. The outcome variable was low SWB (LSWB). The study used univariate and bivariate analysis for reporting the initial results. Further, the study employed the structural equation modeling (SEM) technique using maximum likelihood estimation (MLE) procedure to estimate the covariance matrix.

**Results:**

Overall, about 27% of older adults reported LSWB. Reporting LSWB was more prevalent among older adults who had no income (30.8%) and those who had income but not sufficient to fulfil their basic needs (39.4%, *p* < 0.001). The prevalence of reporting LSWB was significantly higher among older adults who had no asset ownership (36.5%, *p* < 0.001) than those who had asset ownership. The path from the SEM shows that LSWB and SES are negatively related to each other. Moreover, LSWB had significant negative relationship with independence (β = -0.032, *p* < 0.001) and social capital (β = -0.020; *p* < 0.001). In addition, results found a positive relationship between SES and independence (β = 0.019; *p* < 0.001), SES and social capital (β = 0.016; *p* < 0.001), and independence and social capital (β = 0.033; *p* < 0.001).

**Conclusions:**

The findings highlight that higher SES, good physical functioning as well as favorable social capital are interdependent factors of late-life wellbeing and a multidimensional approach in policymaking can ensure a successful and active ageing among older Indian adults.

**Supplementary Information:**

The online version contains supplementary material available at 10.1186/s12889-022-14215-4.

## Introduction

Aging population across world countries and in low- and middle-income countries in particular poses wide-ranging health, social, and economic challenges, both current and future. India is projected to surpass China as the world’s most populous country during 2023 [1] with a rapidly growing proportion of older persons that requires improvement of social security and long-term care systems in terms of ensuring a successful aging process [2].

Researchers have used different measures to understand who is happy in their later years of life and what contributes to their happiness. Subjective well-being (SWB) is documented as consisting of three aspects of the presence of positive as well as negative affect and life satisfaction [3]. A substantial body of research has considered SWB as an important factor in successful ageing and an indicator of efficacy in old age [4–6]. Factors contributing to the late-life wellbeing have been extensively elaborated in past studies. Such factors include the socio-demographic characteristics, personality, economic conditions, health situations and goals and life choices of older people [7–9].

The correlates of SWB in a multidimensional point of view combined with diverse aspects such as social integration, functional skills, education, income and other socioeconomic conditions, have been less explored [10–12]. Considering the relationship between advancing age and SWB, some authors have demonstrated that physical health with a better functionality is essential to facilitate positive self-perception as individuals age [13–15]. Recent studies have shown that different types of living arrangements and the feelings about it directly [16–18], and indirectly through social support function [19], play an important role in predicting SWB and life satisfaction for older adults.

### Relationship between SES, functional independence and social capital

Furthermore, research showed that a low SES is associated with more disadvantages and increased chronic illnesses and functional disabilities [20–22]. A recent study among older Indian adults suggested that socioeconomic disadvantages are positively associated with old age physical frailty [23]. On the other hand, a higher SES was found to be negatively associated with physical disability, frailty and mortality [24, 25].

Similarly, social capital has been studied from different view-points including available support networks, social content and involvements, and self-perceived support such as being in a marital union and co-residential living arrangements [17, 26–28]. Also, studies found that a detrimental effect of low SES on social engagement results in increased feeling of loneliness among older adults [26, 29], whereas, those with more socioeconomic resources are advantaged with better opportunities to increase their social networks [30]. Besides, social support networks and physical exercise have been shown to be protective against functional difficulties, in turn reducing the chances of mental illnesses [31–33].

### SWB as a function of SES, functional independence and social capital

Studies on the link between SES and life satisfaction among aged populations has drawn a great deal of attention in the literature [34]. Recent research highlighted that SES is an important contributing factor to the well-being of older adults [35, 36]. Some researchers argue that the direct impact of SES on SWB may be mediated by community resources [37, 38]. Studies showed that social support is associated both with SES and health and theoretically falls on the causal pathway linking the two with SES influencing social capital which in turn influences subjective health status [39, 40]. Although there is general agreement that SES–SWB relations are stronger within low income countries [41], the empirical evidence is scarce in developing countries.

Further, social support is shown to be an essential factor for ensuring psychological wellbeing among older individuals irrespective of resource-poor or resource-rich care settings [42–44]. On the other hand, unsupportive and unfriendly relationships and social networks can lead to poor social interactions, distress, and disappointment, all of which affect the older adults’ perception of well-being [36, 45]. SES-related differences in structural and functional forms of social support was observed in a study as contributing to poor health and increased mortality among older population [46].

Since socioeconomic-related measures might not alone be able to capture all influences affecting subjective health, particularly in less wealthy countries like India, examination of individual role of SES, functional independence, and social capital contributes to the understanding of the bigger picture regarding the older adults' wellbeing. This study aimed to explore the possible pathways from SES, functional independence, and social capital towards SWB in a representative sample of older adults in India.

## Methods

### Data

The current study used data from the “Building a Knowledge Base on Population Aging in India” (BKPAI) survey, which was conducted in seven Indian states in 2011 [47]. The Institute for Social and Economic Change (ISEC) in Bangalore, the Tata Institute for Social Sciences (TISS) in Mumbai, the Institute for Economic Growth (IEG) in New Delhi, and the United Nations Population Fund (UNFPA) in New Delhi all funded to the study [47]. The survey gathered information on several socioeconomic and health aspects of ageing in households with residents aged 60 and higher [47]. North India, South India, Western India, and Eastern India were among the seven states where data was gathered to represent India's different regions [47]. In the north, Punjab and Himachal Pradesh were located, in the south, Kerala and Tamil Nadu were located, in the east, Orissa and West Bengal were located, and in the west, Maharashtra was located [47].

The probability proportional to size (PPS) sampling approach was used to choose the Primary Sampling Units (PSUs), and older people's residences were selected through systematic sampling inside each main sample unit (PSUs) [47]. In metropolitan areas, a similar approach was applied, and then a sample of people from all seven states was chosen [47]. A total of 9850 people aged 60 and up were questioned from 8329 households. The sample of 9231 older people were included in the study after all pre-analytical processes were completed, such as deleting missing data (390 cases) and outliers (229 cases). All methods were carried out in accordance with relevant guidelines and regulations.

### Variable description

#### Outcome variable

The questions used to assess SWB were 1) Do you feel your life is interesting? 2) Compared with the past, do you feel your present life is better? 3) On the whole, how happy are you with the kind of things you have been doing in recent years? 4) Do you think you have achieved in your life the standard of living and the social status that you had expected? 5) How do you feel about the extent to which you have achieved success and are getting ahead? 6) Do you normally accomplish what you wanted to accomplish? 7) Do you feel you can manage situations even when they do not turn out to be as expected? 8) Do you feel confident that in case of a crisis (anything that substantially upsets your situation in life) you will be able to handle it or face it boldly? 9) The way things are going now; do you feel confident in coping with your future? The responses were 1 “Very much” 2 “To some extent” and 3 “Not so much”. The final coding was done as 0 “very much” and 1 “To some extent/Not so much”. And a final score was developed by summing them and it ranged from 0 to 9 [16]. Thus, the SWB was having a scale of 0 to 9 and has been treated as latent variable in the structural equation modeling and was categorized as 0 “high” (representing 6 + scores) and 1 “low” (representing score 5 and less) (LSWB) during bivariate analysis [16].

#### Exposure variables

1. Self-perceived income sufficiency was coded as no income, has income and fully sufficient, has income and partially sufficient, and has income and not sufficient.
2. Working status was coded as never worked, currently working and retired.
3. Received pension was coded as no and yes.
4. Asset ownership was coded as no and yes.
5. Sex was coded as men and women.
6. Co-residing with children was recoded as no and yes.
7. Age was recoded as 60–69 years, 70–79 years and 80 + years [48].
8. Educational status was recoded as no education, below five years, 6–10 years and 11 + years.
9. Marital status was recoded as not in union and currently in union [16].
10. Ability to do activities of daily living (ADL) was having a scale of 0 to 6 where higher the score higher the independence. It was coded as 0, which represents complete independence and 5 and less as 1, which represents not completely independent to do activities of daily living (Cronbach alpha: 0.93) [17].
11. Ability to do instrumental activities of daily living (IADL) was having a scale of 0 to 8, where higher the score higher the independence. A score of 6 + was categorized as 0 representing high IADL and score of 5 and less was recoded as 1 representing low IADL [49].
12. Disability was coded as no and yes. Disabilities included disability of vision, hearing, memory, walking, teeth (chewing), and speaking. Full and partial disability was clubbed as 1 “yes” and neither of any was clubbed as 0 “no”.
13. Five questions for involvement in the community were asked and were used to create a variable to measure community involvement. The score developed ranges from 0 to 5, and score of 0 was coded as 0 “no community involvement” and score 1 to 5 was coded as 1 “community involvement”.
14. Trust over someone was assessed using the question “do you have someone you can trust and confide in?” and was recoded as 0 “yes” and 1 “no”.
15. Decision making power was recoded as no role, partial decision making (with someone else) and absolute role (alone) [36]. This variable was created using the question, “Who usually makes the decisions: you alone, or with your spouse, with your children, or with others, on the following issues, a) marriage of son/daughter, b) buying and selling of property, c) buying other household items, d) gifts to daughters, grandchildren, other relatives, e) education of children, grandchildren and f) arrangements of social and religious events?” (Cronbach’s alpha: 0.88)
16. Economic violence was coded as no and yes. The variable was created using the question, “What kind of abuse did you face?”
17. Caste was recoded as Scheduled Caste (SC), Scheduled Tribe (ST), Other Backward Class (OBC) and other. The SC includes a group of the population that is socially segregated and financially/economically by their low status as per Hindu caste hierarchy. The SCs and STs are among the most disadvantaged socio-economic groups in India. The OBC is the group of people who were identified as “educationally, economically and socially backward”. The “other” caste category is identified as having higher social status [50].
18. Religion was recoded as Hindu, Muslim, Sikh and others.
19. Wealth index was recoded as poorest, poorer, middle, richer and richest. The wealth index drawn based on the BKPAI survey is based on the following 30 assets and housing characteristics: household electrification; drinking water source; type of toilet facility; type of house; cooking fuel; house ownership; ownership of a bank or post-office account; and ownership of a mattress, a pressure cooker, a chair, a cot/bed, a table, an electric fan, a radio/transistor, a black and white television, a color television, a sewing machine, a mobile telephone, any landline phone, a computer, internet facility; a refrigerator, a watch or clock, a bicycle, a motorcycle or scooter, an animal-drawn cart, a car, a water pump, a thresher, and a tractor [47]. The range of index was from poorest to the richest i.e. ranging from lowest to the highest [47].
20. Place of residence was recoded as urban and rural.
21. State was recoded as Himachal Pradesh, Punjab, West Bengal, Odisha, Maharashtra, Kerala and Tamil Nadu.

### Statistical analysis

Univariate and bivariate analyses were used to report the characteristics of the data and the prevalence of LSWB by selected background characteristics. Chi-square test was conducted to check if there were significant associations between LSWB and background characteristics. Cronbach's alpha was used to assess the indicators' consistency, which is an important step in the SEM framework for determining data quality. Finally, using the SEM approach and the Maximum Likelihood Estimation (MLE) process, the covariance matrix was calculated (Supplementary Table S1). The model-fit-indices, the statistical significance of the parameter estimates, and the effect-size and its direction are all criteria that are commonly utilized in the evaluation of SEM [51]. *Svyset* command was used to control the analysis for complex survey design. The individual weights were also used for computing the estimates and make them nationally representative.

#### Reliability and validity analysis

The study employed the standard approach used in prior studies to assess the consistency and stability of measurement variables [51]; a Cronbach’s alpha [52] above 0.70 was considered an acceptable level of the reliability analysis. All the measurement variables loaded high on each latent construct and were considered for the final analysis (Supplementary Table S3). Supplementary Table S2 provides the description of Eigenvalue.

#### Model fit criteria

There are few criteria which were recommended for determining the model fit in SEM. However, the model fit criteria vary across the studies, and the chi-square was observed as the conventional and the most used measure for assessing the model fit [53]. However, chi-square is always sensitive to sample size [54]. Therefore, the root-mean square error of approximation (RMSEA) which is non-sensitive to sample size is often a recommended technique [55]. Usually, the traditional level of RMSEA below 0.08 indicates a better fit model [56]. Additionally, the other fit indices are comparative fit index (CFI) and goodness of fit index (GFI) [57].

### Model evaluation

#### Fit statistics

**Fig. 1:**
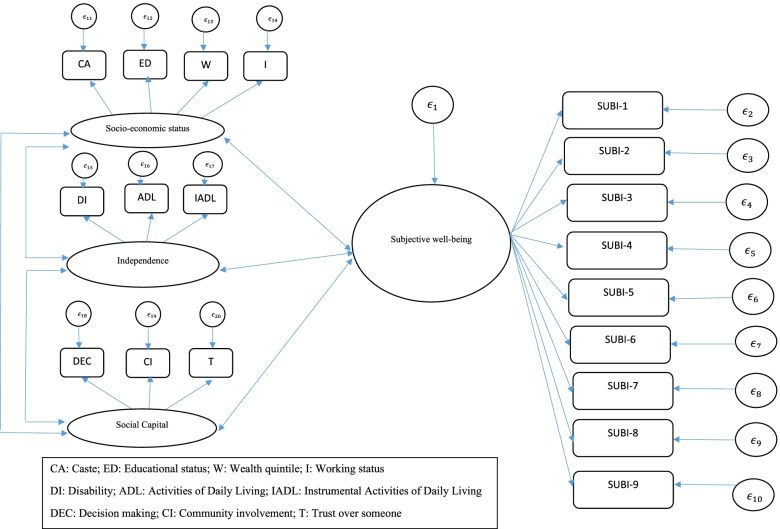
SEM model

SEM path diagram has been generated through Stata 15 software that had standardized estimates and goodness-of-fit indices (Fig. 1/Table 3). The chi-square test statistic was 4387.67, df = 146, *p* < 0.001; but because the chi-square test is very sensitive to sample size (*n* = 9231), we considered the supplementary goodness-of-fit index- RMSEA, which indicated a good fit at 0.046; acceptable level of RMSEA used in this study was < 0.05. Other fit statistics such as CFI, Tucker-Lewis Index (TLI) and Standardized Root Mean Square Residual (SRMR) are considered as model indices [58]. The value of CFI and TLI ≥ 0.95 is considered to be best fit model; however, the value above 0.90 is also acceptable [59]. SRMR is defined as the standardized difference between the observed correlation and the predicted correlation. It is a positively biased measure, and that bias is greater for small N and for low degree freedom studies. The SRMR has no penalty for model complexity. A value less than 0.08 is generally considered a good fit [60]. The SRMR value in this study was 0.04.

## Results

### Socio-economic and demographic profile of study participants in India (*Table **1*)

**Table 1:** Socio-economic and demographic factors among older adults

Background characteristics	Sample	Percentage
**Individual factors**		
**Self-perceived income sufficiency**		
No income	3,967	43.0
Has income and fully sufficient	2,168	23.5
Has income and partially sufficient	2,433	26.4
Has income and not sufficient	663	7.2
**Working Status**		
Never worked	6,212	67.3
Currently working	2,223	24.1
Retired	796	8.6
**Received pension**		
No	6,447	69.8
Yes	2,784	30.2
**Asset ownership**		
No	1,630	17.7
Yes	7,601	82.3
**Sex**		
Men	4,372	47.4
Women	4,859	52.6
**Co-residing with children**		
No	2,738	29.7
Yes	6,493	70.3
**Age group (in years)**		
60–69	5,704	61.8
70–79	2,536	27.5
80 +	991	10.7
**Educational status**		
No education	4,684	50.7
Below 5 years	1,900	20.6
6 to 10 years	2,086	22.6
11 + years	562	6.1
**Marital status**		
Not in union	3,649	39.5
Currently in union	5,582	60.5
**Difficulty in IADL**		
No	4,008	43.4
Yes	5,223	56.6
**Difficulty in ADL**		
No	8,541	92.5
Yes	690	7.5
**Disability**		
No	2,519	27.3
Yes	6,712	72.7
**Community involvement**		
No	1,896	20.5
Yes	7,335	79.5
**Trust over someone**		
No	7,652	82.9
Yes	1,579	17.1
**Contextual factors**		
**Decision making power**		
No role	512	5.6
Partial decision making	2,218	24.0
Absolute role	6,501	70.4
**Economic violence**		
No	8,781	95.1
Yes	450	4.9
**Caste**		
Scheduled Caste	1,911	20.7
Scheduled Tribe	515	5.6
Other Backward Class	3,364	36.4
Others	3,441	37.3
**Religion**		
Hindu	7,324	79.3
Muslim	651	7.1
Sikh	870	9.4
Others	386	4.2
**Wealth status**		
Poorest	2,169	23.5
Poorer	2,029	22.0
Middle	1,913	20.7
Richer	1,720	18.6
Richest	1,399	15.2
**Place of residence**		
Rural	6,827	74.0
Urban	2,404	26.0
**State**		
Himachal Pradesh	1,471	15.9
Punjab	1,279	13.9
West Bengal	1,128	12.2
Orissa	1,454	15.8
Maharashtra	1,229	13.3
Kerala	1,341	14.5
Tamil Nadu	1,330	14.4
**Total**	9,231	100

Table 1 presents the socio-economic and demographic profile of study participants. Nearly 43% of older adults reported that they did not have income to fulfil their basic needs whereas only 24% of older adults had sufficient income to fulfil their basic needs. Around one-fourth of older adults were currently working, 30% were receiving pension, and majority of older adults reported asset ownership (82.3%). About 70% of older adults were co-residing with children, half of older adults had no education, and 60% were currently in a marital union. More than half of the older adults had difficulty in IADL and about eight per cent of older adults reported difficulty in ADL. About three-fourth of older adults suffered from disability, 80% reported community involvement and only 17% of older adults had trust over someone. Nearly 70% of older adults had absolute role in decision making power and about five per cent of older adults faced economic violence in the household.

### Percentage of older adults suffered from low subjective well-being (LSWB) in India (*Table **2*)

**Table 2:** Percentage of older adults suffering from low subjective well-being

Background characteristics	Low subjective well-being	
**Individual factors**	**(%)**	***p*** **-value**
**Self-perceived income sufficiency**		< 0.001
No income	30.8	
Has income and fully sufficient	15.6	
Has income and partially sufficient	26.6	
Has income and not sufficient	39.4	
**Working Status**		< 0.001
Never worked	30.4	
Currently working	23.5	
Retired	7.0	
**Received pension**		< 0.001
No	27.6	
Yes	24.6	
**Asset ownership**		< 0.001
No	36.5	
Yes	24.6	
**Sex**		< 0.001
Men	23.9	
Women	29.3	
**Co-residing with children**		< 0.001
No	29.9	
Yes	25.4	
**Age group (in years)**		< 0.001
60–69	23.2	
70–79	30.0	
80 +	38.7	
**Educational status**		< 0.001
No education	35.5	
Below 5 years	24.0	
6 to 10 years	13.9	
11 + years	10.3	
**Marital status**		< 0.001
Not in union	32.9	
Currently in union	22.7	
**Difficulty in IADL**		< 0.001
No	16.1	
Yes	34.9	
**Difficulty in ADL**		< 0.001
No	24.5	
Yes	54.8	
**Disability**		< 0.001
No	15.8	
Yes	30.8	
**Community involvement**		< 0.001
No	40.2	
Yes	23.5	
**Trust over someone**		< 0.001
No	23.3	
Yes	42.9	
**Contextual factors**		
**Decision making power**		< 0.001
No role	55.9	
Partial decision making	33.0	
Absolute role	22.3	
**Economic violence**		< 0.001
No	25.8	
Yes	45.0	
**Caste**		< 0.001
Scheduled Caste	33.8	
Scheduled Tribe	35.1	
Other Backward Class	27.8	
Others	20.5	
**Religion**		< 0.001
Hindu	28.5	
Muslim	29.9	
Sikh	12.1	
Others	21.6	
**Wealth status**		< 0.001
Poorest	47.3	
Poorer	32.4	
Middle	21.1	
Richer	14.7	
Richest	9.3	
**Place of residence**		< 0.001
Rural	28.3	
Urban	22.4	
**State**		< 0.001
Himachal Pradesh	15.1	
Punjab	11.4	
West Bengal	48.3	
Orissa	35.3	
Maharashtra	34.3	
Kerala	14.8	
Tamil Nadu	31.7	
**Total**	26.7	

*p*-value are based on chi-square test

Table 2 presents the percentage of older adults reporting LSWB*.* Overall, about 27% of older adults reported LSWB. LSWB was more prevalent among older adults who had no income (30.8%) and those who had income but not sufficient to fulfil their basic needs (39.4%, *p* < 0.001). Older adults who never worked reported significantly more LSWB (30.4%, *p* < 0.001). The prevalence of LSWB was significantly more among older adults who had no asset ownership (36.5%, *p* < 0.001) than those who had asset ownership. LSWB was significantly higher among women than men (29.3% vs. 23.9%, *p* < 0.001). Older adults who were co-residing with children reported less LSWB compared to their counterparts (25.4%, *p* < 0.001). Education and wealth index had negative association with LSWB. For instance, as the level of education and wealth increased, the prevalence of LSWB decreased. The prevalence of LSWB was significantly higher among older adults who had difficulty in IADL (34.9%, *p* < 0.001), difficulty in ADL (54.8%, *p* < 0.001), suffered from disability (30.8%, *p* < 0.001), had no community involvement (40.2%, *p* < 0.001), and those who had trust over someone (42.9%, *p* < 0.001). Older adults who had no role in decision making reported higher prevalence of LSWB (55.9%, *p* < 0.001) whereas those faced violence in the household reported more LSWB (45%, *p* < 0.001) compared to their counterparts. The prevalence of LSWB was significantly higher in rural areas than urban counterparts (28.3% vs. 22.4%, *p* < 0.001).

#### Measurement variables analysis

The study reported multivariate standardized parameter estimates and the estimates corresponded to the effect size, which indicated that all the measurement variables significantly defined each respective latent variable at *p*-value < 0.001 (Table 3, Fig. 1). All the observed variables were included in the structural model. Standardized coefficients are model parameter estimates based on the analysis of standardized data, in the sense that all variables are supposed to have unit variance. Standardized data are affected less by the scales of measurement and can be used to compare the relative impact of variables that are incommensurable (i.e., measured in different units on the same/different scales).

**Table 3:** Multivariate standardized parameter estimates (**β)**, *p*-value and 95% confidence interval of the measurement variables in the structural equation model

Codes	Indicators	β (95% CI)	*p*-value
**LSWB**	**Subjective well-being (α = 0.85)**		
SUBI1			
SUBI2		1.24 (1.20,1.29)	< 0.001
SUBI3		1.27 (1.22,1.31)	< 0.001
SUBI4		1.10 (1.05,1.14)	< 0.001
SUBI5		1.15 (1.11,1.20)	< 0.001
SUBI6		1.03 (0.98,1.07)	< 0.001
SUBI7		1.14 (1.10,1.19)	< 0.001
SUBI8		1.24 (1.19,1.28)	< 0.001
SUBI9		1.13 (1.09,1.18)	< 0.001
**SES**	**Socio-economic status (α = 0.67)**		
Caste			
Education		1.75 (1.60,1.89)	< 0.001
Wealth status		1.75 (1.61,1.89)	< 0.001
Working status		0.36 (0.27,0.45)	< 0.001
**I**	**Independence (α = 0.78)**		
IADL			
ADL		0.44 (0.40,0.48)	< 0.001
Disability		0.43 (0.37,0.49)	< 0.001
**SC**	**Social capital (α = 0.88)**		
Community involvement			
Decision making power		0.48 (0.43,0.52)	< 0.001
Trust over someone		0.64 (0.57,0.72)	< 0.001
**Model Fit Statistic**			
Chi-Square	0.001		
**RMSEA**	0.046		
CFI	0.880		
TLI	0.860		
SRMR	0.042		
CD	0.965		

*α*: Cronabach alpha, *CD* Coefficient of determination, *CFI* Comparative Fit Index, *RMSEA* Root Mean Square Error of Approximation, *SRMZ* Standardized Root Mean Square Residual, *TLI* Tucker–Lewis index

**Table 4:** Multivariate standardized covariance coefficient (β), *p*-value and 95% confidence interval of the estimated structural equation model

	**β (95% CI)**	***p*-value**
Cov (LSWB, SES)	-0.019 (-0.020,-0.017)	(0.001)
Cov (LSWB, I)	-0.032 (-0.034,-0.029)	(0.001)
Cov (LSWB, SC)	-0.020 (-0.022,-0.018)	(0.001)
Cov (SES,I)	0.019 (0.016,0.021)	(0.001)
Cov (SES,SC)	0.016 (0.014,0.018)	(0.001)
Cov (I,SC)	0.033 (0.029,0.036)	(0.001)

#### Multivariate structural model analysis

The path from SEM shows that LSWB and SES are negatively related to each other (Table 4). For instance, one-unit increase in SES, decreased by 0.019 unit in LSWB. Moreover, LSWB had significant negative relationship with independence (β = -0.032, *p* < 0.001) and social capitals (β = -0.020; *p* < 0.001). In addition, results found a positive relationship between SES and independence (β = 0.019; *p* < 0.001), SES and social capital (β = 0.016; *p* < 0.001), and independence and social capitals (β = 0.033; *p* < 0.001). Additionally, Fig. 1 represents the SEM model.

## Discussion

This study, by empirically examining the associations of SES, physical functioning and social capital with SWB, adds to the existing studies on successful ageing considering SWB as one of the important indicators [61–63], by relating three objective measures to a subjective construct. It has shown multiple direct pathways among different dimensions of SWB.

Considering the relationship between socio-demographic factors and LSWB, findings from the current study revealed that persons in the oldest old age group (80 +) had a higher level of SWB than their younger counterparts. This is compatible with a phenomenon known as the paradox of ageing that shows the reduced emotional reactions to the negative situations influenced by shifts in the preferred strategies and goal priorities by advancing age [64, 65]. Therefore, older adults in higher age groups are able to maintain positive psychological well-being which leads to greater levels of SWB among them. On the other hand, a few longitudinal studies found a decrease in life satisfaction in oldest old age groups [66, 67]. Although some authors have argued that the task of evaluating SWB prompts individuals to focus on the objective circumstances such as wealth index and educational level [68], recent studies have shown an independent association of perceived income adequacy and late life wellbeing [69–71]. Consistently, older participants with a self-perceived income insufficiency had higher LSWB in the present study. Further, a higher educational level in the present study was found to be protective against LSWB, as shown in the earlier studies [72, 73].

Multivariate analysis also has shown a negative association of SES construct with LSWB, in parallel to findings from earlier studies showing the direct and indirect effects of SES and SWB [37]. This also supports findings from multiple studies that have suggested that LSWB among older individuals in economically depressed areas could be improved through some interventions addressing the socioeconomic disadvantages [74–77]. Moreover, due to the stronger SES-SWB associations in low income countries compared to developed nations [78], such interventions would benefit older adults in the country to achieve higher wellbeing scores.

The findings of this study also demonstrated that functional independence in later years of life is related to SWB. Older adults with lower independence (in ADL and IADL functioning and with disability) had higher LSWB. Similar findings have also been observed in other studies [79, 80]. The results of this study are also consistent with the findings of several studies that have shown the positive effects of a better functional health on wellbeing, including the enhancement of life satisfaction [81]. Thus, as evident from earlier studies as well, improving functional health status could be considered as one means by which government can improve the SWB of their senior citizens [82, 83]. Again, consistent with previous studies that revealed that lack of social contact is strongly positively associated with SWB [84], we found a negative association between the construct of social capital (with higher decision making power, community involvement and having someone to be trusted) and LSWB. This suggests that an active social network and feelings of companionship are important in contributing to satisfaction in life among older people. The findings of the present study also demonstrate the importance of social influences on later life SWB and suggest a need for further investigation of possible mediating factors so that the pathways from SES through measures of social engagement to SWB would be clarified.

Furthermore, the present analysis also confirms other three important associations between SES and functional independence, SES and social capital and functional independence and social capital. It revealed that older people with a higher SES had a better functionality and greater social capital, whereas, those with a better functional health had higher chances of having more social capital. These paths should be further investigated as mediating in the SES-SWB association.

### Research, practice and policy implications

The current findings have implications for future research, and they can be applied to policies and programs for older individuals in the country. Lacking in the literature is studies that address the positive effects of social support and social networks on specific indicators of successful aging, specifically in low-resource settings like India. We call for more research to focus on the relationships between socioeconomic, health and wellbeing indicators among the growing older subpopulation in these countries. It is also important for those who are health practitioners and those who are policymakers to ensure that older adults receive the care and support they need, not only in regard to their physical and functional needs but also their mental well-being. This includes increasing their feeling of usefulness which is shown to be associated with improved physical functioning [85], by enhancing their community participation and involving them in household decision making.

The current findings also support the previous evidence on the possibility of offsetting the negative changes of old age such as physical health deficits and functional disability by super-imposing the psychosocial resources such as improved trust over someone and household/social engagement [61]. Moreover, social workers who work with older adults need to focus on social support systems, including the quality and quantity of people in their network. It also seems necessary to take the SES and access to resources into account, which are relevant to SWB. In this regard, it is important to develop programs and interventions that assist older adults belonging to poor socioeconomic background in utilizing the resources and ensure an equal wellbeing for them.

### Limitations and strength

The dataset for this study was from a cross-sectional study. Hence, data were collected at one point in time and only cross-sectional correlation statistics were utilized in this study; thus, no definite statement on causality can be made. Further studies with longitudinal research design would be more helpful to measure phenomena that changed over time and understand the directionality. Also, the data were obtained exclusively through self-reports from the older participants which might cause reporting and recall bias. Future research could use a mixed-method design including observational data and reports from family members. Finally, the excluded sample due to incomplete data or being outliers (*n* = 619) was belonging to poor socioeconomic strata which might bias the results and influence the representativeness and the generalizability of the current findings. Despite these limitations, this study adds to the literature by investigating the corresponding determinants of SWB. The strength of the present study rests in the number and range of covariates considered, the use of a latent variable modeling technique to control for measurement error and with the insights that it provides into the systematic relationships between SES, social and functional health, and wellbeing. And the findings reinforce the importance of further investigating indirect pathways of influence in relation to wellbeing among older adults.

## Conclusion

The current analysis helps to orient researchers in gerontology by contextualizing different kinds of determinants and showing how more general factors are closely inter-related. This leads to several insights regarding the need for further research in relation to the wellbeing of older adults. The findings highlight that higher SES, good physical functioning as well as favorable social capital are possible interdependent factors of late-life wellbeing and a successful ageing. They also revealed that by working on different pathways of several objective circumstances, multidimensional policies can lead to successful ageing. Again, this study suggests the need for further empirical studies to improve understanding of the primary mechanism of achieving late-life wellbeing and a successful ageing in a country where population aging is increasingly severe.

## Supplementary Information

**Additional file 1: Supplementary Table S1.** Correlation matrix. **Supplementary Table S2.** Description of Eigenvalue. **Supplementary Table S3.** Factor loadings of the latent variable.

## Data Availability

The study utilizes a secondary data which is available on request from director@isec.ac.in at http://www.isec.ac.in/.
