# Supplementary material for: How socioeconomic status, social capital and functional independence are associated with subjective wellbeing among older Indian adults? A structural equation modeling analysis
Source: BMC Public Health. 2022 Sep 30;22:1836. doi: 10.1186/s12889-022-14215-4 (PMC9523926; doi:10.1186/s12889-022-14215-4)
Supplement: Supplementary file 1 — Additional file 1: Supplementary Table S1. Correlation matrix. Supplementary Table S2. Description of Eigenvalue. Supplementary Table S3. Factor loadings of the latent variable. [file 12889_2022_14215_MOESM1_ESM.docx]

| **Supplementary table S1.** Correlation matrix | | | | | | | | | | |
| --- | --- | --- | --- | --- | --- | --- | --- | --- | --- | --- |
|  | Education | Wealth status | Caste | Working status | Community involvement | Trust over someone | Decision-making power | ADL | IADL | Disability |
|  |  |  |  |  |  |  |  |  |  |  |
| Education | 1.000 |  |  |  |  |  |  |  |  |  |
| Wealth status | 0.330 | 1.000 |  |  |  |  |  |  |  |  |
| Caste | 0.256 | 0.272 | 1.000 |  |  |  |  |  |  |  |
| Working status | 0.137 | -0.013 | -0.042 | 1.000 |  |  |  |  |  |  |
| Community involvement | 0.153 | 0.116 | 0.038 | 0.086 | 1.000 |  |  |  |  |  |
| Trust over someone | 0.117 | 0.211 | 0.074 | 0.037 | 0.126 | 1.000 |  |  |  |  |
| Decision-making power | 0.109 | 0.099 | 0.023 | 0.117 | 0.208 | 0.099 | 1.000 |  |  |  |
| ADL | 0.062 | 0.024 | -0.001 | 0.094 | 0.209 | 0.057 | 0.135 | 1.000 |  |  |
| IADL | 0.264 | 0.136 | 0.095 | 0.141 | 0.182 | 0.098 | 0.149 | 0.215 | 1.000 |  |
| Disability | 0.057 | 0.018 | 0.017 | 0.006 | 0.004 | -0.010 | 0.042 | 0.147 | 0.118 | 1.000 |

| **Supplementary table S2.** Description of Eigenvalue | |
| --- | --- |
| **Factors** | **Eigenvalue** |
|  |  |
| Factor1 | 3.57 |
| Factor2 | 0.33 |
| Factor3 | 0.12 |
| Factor4 | -0.02 |
| Factor5 | -0.06 |
| Factor6 | -0.08 |
| Factor7 | -0.14 |
| Factor8 | -0.16 |
| Factor9 | -0.18 |

| **Supplementary table S3.** Factor loadings of the latent variable | | | | |
| --- | --- | --- | --- | --- |
| **Variable** | **Factor1** | **Factor2** | **Factor3** | **Uniqueness** |
|  |  |  |  |  |
| SUBI1 | 0.6086 | -0.2279 | -0.0847 | 0.5705 |
| SUBI2 | 0.6605 | -0.2913 | -0.0484 | 0.4765 |
| SUBI3 | 0.6648 | -0.2129 | -0.0133 | 0.5125 |
| SUBI4 | 0.6064 | -0.0211 | 0.1583 | 0.6068 |
| SUBI5 | 0.6325 | 0.0298 | 0.1759 | 0.5681 |
| SUBI6 | 0.5699 | 0.1347 | 0.1365 | 0.6385 |
| SUBI7 | 0.6248 | 0.2566 | -0.0345 | 0.5427 |
| SUBI8 | 0.6596 | 0.2155 | -0.1471 | 0.4969 |
| SUBI9 | 0.6368 | 0.1372 | -0.1164 | 0.5621 |
